# Dynamic Recrystallization Behavior and Corrosion Resistance of a Dual-Phase Mg-Li Alloy

**DOI:** 10.3390/ma11030408

**Published:** 2018-03-09

**Authors:** Gang Liu, Wen Xie, Guobing Wei, Yan Yang, Junwei Liu, Tiancai Xu, Weidong Xie, Xiaodong Peng

**Affiliations:** 1 College of Materials Science and Engineering, Chongqing University, Chongqing 400044, China; 20140901001@cqu.edu.cn (G.L.); 20160913145@cqu.edu.cn (W.X.); Yanyang@cqu.edu.cn (Y.Y.); xtc@cqu.edu.cn (T.X.); wdxie@cqu.edu.cn (W.X.); pxd@cqu.edu.cn (X.P.); 2 National Engineering Research Center for Magnesium Alloys, Chongqing 400044, China; 3 School of mechatronics engineering, Zhengzhou University of Aeronautics, Zhengzhou 450015, China; ljeiloveyou@163.com

**Keywords:** hot deformation, dynamic recrystallization, Mg-Li alloy, corrosion resistance

## Abstract

The hot deformation and dynamic recrystallization behavior of the dual-phase Mg-9Li-3Al-2Sr-2Y alloy had been investigated using a compression test. The typical dual-phase structure was observed, and average of grain size of as-homogenized alloy is about 110 µm. It mainly contains β-Li, α-Mg, Al_4_Sr and Al_2_Y phases. The dynamic recrystallization (DRX) kinetic was established based on an Avrami type equation. The onset of the DRX process occurred before the peak of the stress–strain flow curves. It shows that the DRX volume fraction increases with increasing deformation temperature or decreasing strain rate. The microstructure evolution during the hot compression at various temperatures and strain rates had been investigated. The DRX grain size became larger with the increasing testing temperature or decreasing strain rate because the higher temperature or lower strain rate can improve the migration of DRX grain boundaries. The fully recrystallized microstructure can be achieved in a small strain due to the dispersed island-shape α-Mg phases, continuous the Al_4_Sr phases and spheroidal Al_2_Y particles, which can accelerate the nucleation. The continuous Al_4_Sr phases along the grain boundaries are very helpful for enhancing the corrosion resistance of the duplex structured Mg-Li alloy, which can prevent the pitting corrosion and filiform corrosion.

## 1. Introduction

Due to the outstanding advantages, such as exceptionally low density (1.35–1.65 g/cm^3^), high specific strength, good electromagnetic shielding performance and excellent formability, the Mg-Li alloys have become promising materials for automobile, aerospace, the military and 3C industry [1,2,3]. According to the Mg-Li binary phase diagram, the alloys can be divided into three groups on account of variety of lithium contents and crystal structures. When the lithium content in Mg-Li alloys is less than 5.5 wt %, the alloy is mainly composed of the α-Mg phases with hexagonal close-packed crystal (hcp) structure. While the lithium content in Mg-Li alloys is more than 11.5 wt %, the alloy is mainly composed of the β-Li phases with body-centered cubic crystal (bcc) structure. The Mg-Li alloys containing 5.5–11.5% (wt %) Li exhibit a duplex phase structure, consisting of α-Mg (hcp) and β-Li (bcc) phases. Mg-Li alloys have better formability compared to other Mg based alloys due to the decrease of c/a axial ratio. In dual phase Mg-Li alloys, the β-Li phases with bcc structure are more soft and ductile, while the hard α-Mg phases were distributed in the matrix. Hence, Mg-Li alloys with duplex phases usually have better comprehensive mechanical properties (a good combination of strength and elongation, and good super plasticity) than single phase alloys (α-Mg or β-Li). The Mg-9Li and Mg-9Li-X alloys are widely researched, such as Mg-9Li-0.3Y, Mg-9Li-7Al-1Sn, Mg-9Li-5Al-3Sn-1Zn, Mg-9Li-3Al-2.5Sr, Mg-9Li-x(Al-Si)-yTi, and Mg-9Li-3Al alloys by Sn and Y addition [1,2,4,5,6]. These alloys have better comprehensive mechanical properties compared with single phase Mg-Li alloys. Although the dual-phase Mg-Li alloys have some advantages compared with other alloys, the corrosion resistance greatly limits the rapid development and widely commercial applications [7,8]. The lower corrosion resistance of Mg-Li alloys compared to other Mg alloys is ascribed to their sensitive to localized corrosion at the α-Mg/β-Li interfaces [9,10]. Recently, it has been reported that the addition of rare-earth or alkaline earth can enhance the corrosion resistance of Mg-Li alloys [8,11].

The microstructural evolution during deformation at elevated temperatures of Mg alloys is very sensitive to temperature, strain and strain rate [12]. Magnesium alloy is an alloy of low stacking fault energy, and the softening mechanism in the process of thermal deformation is mainly dominated by dynamic recrystallization, which is controlled by the mobility of dislocations. The new recrystallized equiaxed grains will occur during the DRX process, which refined the microstructure and enhanced the strength of alloys. Through adjusting the deformation parameters (i.e., strain rate, temperature, and deformation degree), the DRX process can be easily controlled, and the desired grain size distribution and mechanical properties can be obtained. Thus, study on hot deformation behavior and dynamic recrystallization of magnesium alloys is conducive to optimization of process parameters and improvement of product performance. Although many researchers have studied the microstructural evolution during hot deformation and related recrystallization behavior for Mg-Li alloys, the quantitative relationships between microstructural evolution and thermomechanical history are very few. 

The comprehensive mechanical properties of Mg-9Li-3Al alloy can be improved by addition of Sr and the ultimate tensile strength of 235 MPa and elongation of 19.4% can be achieved, respectively [13]. However, with the Sr increasing, the bulk and inhomogeneous Al_4_Sr will be formed, which is detrimental to the mechanical properties. Thus, yttrium is added due to grain refinement and solution strengthening. The dispersed Al_2_Y particles will increase the stored energy and develop more complex dislocation arrangements, which will increase the driving force for recrystallization.

In this study, the Mg-9Li-3Al-2Sr-2Y alloy with dual phase was designed. First of all, this study will develop quantitative equations to predict the recrystallization behavior based on hot-deformation parameters. Moreover, the effects of Al_4_Sr phases on the corrosion resistance of Mg-9Li-3Al-2Sr-2Y alloy will be discussed.

## 2. Experimental

The Mg-9Li-3Al-2Sr-2Y alloy was prepared in a vacuum resistance furnace. The chemical composition of the experimental alloys was determined by inductively coupled plasma atomic emission spectroscopy (ICP-AES). Actual chemical compositions of experimental alloys are 83.28 Mg, 9.50 Li, 3.05Al, 2.02Sr and 2.15 Y (wt %). When the vacuum degree reaches 1.0 × 10^−2^ Pa, argon is injected into the resistance furnace until it reaches 1.0 × 10^4^ Pa. Then, the melting process is operated under the argon atmosphere, which can keep Mg and Li from evaporation. The melt was stirred and then poured into a cylindrical die. The homogenization process involves heating the billets to 300 °C followed by 4 h hold and air cooling in a vacuum furnace. Before the experiment, the compression specimens were cut from the center of the billet with a cylinder axis parallel to the axial direction of the rod. Then, cylindrical specimens with a diameter of 10 mm and a length of 15 mm were machined by lathe and grinder. On a servo-hydraulic, computer-controlled Gleeble 3500 thermal-mechanical simulator (Gleeble, Poestenkill, NY, USA), the specimens were heated at a rate of 10 °C/s from room temperature to a fixed temperature. The specimens were isothermal compressed to a true strain of 0.4 at the temperatures of 150 °C, 200 °C, 250 °C and 300 °C, and the strain rates of 0.001 s^−1^, 0.01 s^−1^, 0.1 s^−1^ and 1 s^−1^, and then immediately cooled down by chilled water to retain the recrystallized microstructures. All the samples were sectioned parallel to the longitudinal compression axis for microstructures’ observation. The specimens for microstructure observation were polished and etched with 2 vol % nital. The microstructures were observed using an optical microscope (Olympus CX31, Tokyo, Japan) and scanning electron microscope (SEM) (JSM7000F, JEOL, Tokyo, Japan) equipped with an energy dispersive X-ray spectrometer (EDS). An X-ray diffraction (XRD) (DMAX 2400, Rigaku, Tokyo, Japan) measurement was used to analyze the phase constituent of the alloy in the 2*θ* scanning range 20–80° with a 0.02° step size. The open-circuit potential (OCP) test and polarization test were performed on an electrochemical workstation (CS150) (Corrtest, Wuhan, China) using 3.5 wt % NaCl solution. 

## 3. Results and Discussion

### 3.1. Microstructural Characterization

**Figure 1:**
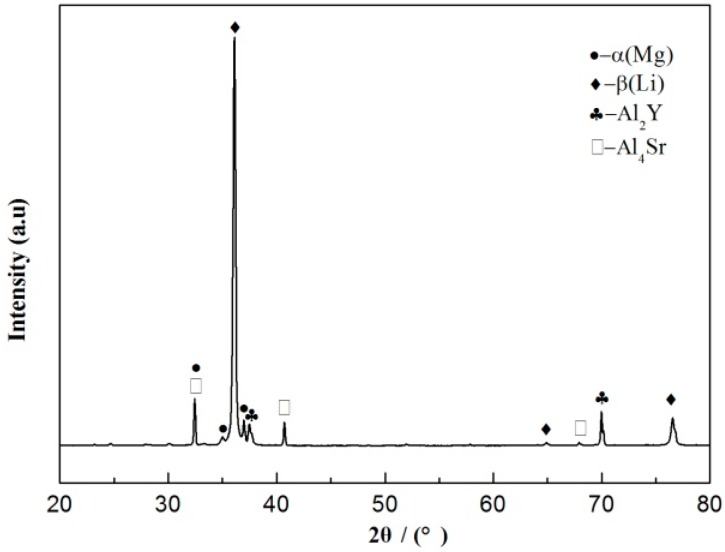
The XRD results of Mg-9Li-3Al-2Sr-2Y alloy.

According to the XRD analysis in Figure 1, the Mg-9Li-3Al-2Sr alloy is mainly composed of β-Li, α-Mg, Al_4_Sr and Al_2_Y.

**Figure 2:**
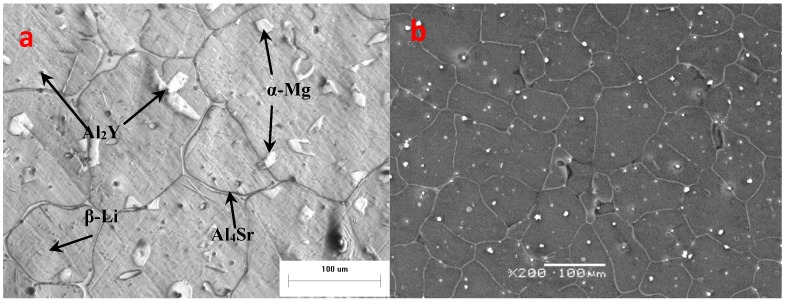
The photographs of as-homogenized alloy: (**a**) The optical microscope (OM) photograph; (**b**) The SEM photograph.

The optical and SEM microstructures of Mg-9Li-3Al-2Sr-2Y alloy are shown in Figure 2. The as-homogenized microstructure of the studied alloy exhibited a duplex phase microstructure, including β-Li matrix plus distributed α-Mg phases with petal-like shapes (Figure 2a). The consecutive network-shaped Al_4_Sr phases are precipitated at the grain boundaries. The granular Al_2_Y phases diffusely distribute on the β-Li matrix and α-Mg phases. The average of grain size is about 110 µm.

**Figure 3:**
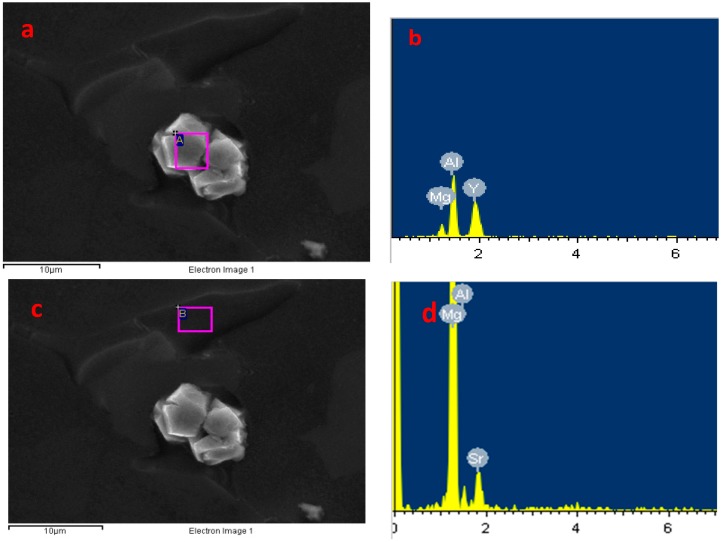
The SEM and Energy Dispersive Spectroscopy (EDS) results of Al_2_Y (**a**,**b**) and Al_4_Sr (**c**,**d**).

As shown in Figure 3, the Al_4_Sr and Al_2_Y phases can be identified by EDS. The intermetallic compounds mainly distributed on the grain boundary are Al_4_Sr phases. The intermetallic compounds mainly dispersed on matrix and α-Mg phases are Al_2_Y phases.

### 3.2. DRX Modeling and Microstructure Evolution

**Figure 4:**
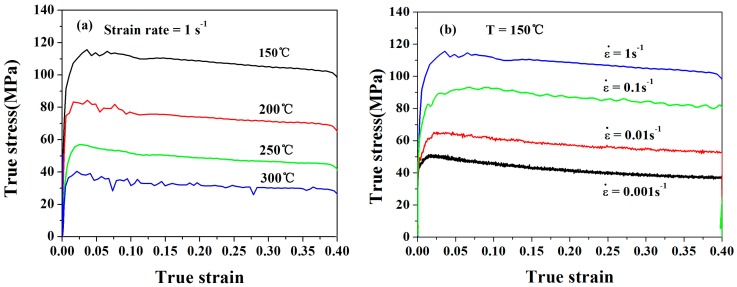
True stress–strain curves for Mg-Li alloy under different deformation conditions: (**a**) The stress–strain curves obtained under strain rate of 1 s^−1^ with different temperatures of 150 °C, 200 °C, 250 °C and 300 °C; (**b**) The stress–strain curves obtained under 150 °C with strain rates of 1 s^−1^, 0.1 s^−1^, 0.01 s^−1^ and 0.001 s^−1^.

The true stress–strain curves obtained during the hot compression of Mg-9Li-3Al-2Sr-2Y alloy in different temperatures and various strain rates are shown in Figure 4. With the deformation rates increasing, the flow stress increases at a fixed deformation temperature. When the strain rate is fixed, the flow stress decreases with higher deformation temperature.

The classical theory of dynamic recrystallization suggests that the dynamic recrystallization needs to meet two conditions: the storage capacity of the grain reaches the maximum value and the dissipation rate drops to the minimum. Y. Bergstrom [14,15] has proposed an equation that has been used to describe dynamic recrystallization for isothermal phase transformation kinetics. The DRX based on flow stress of materials characterized by can be expressed as following equation:

(1) $$X_{DRX}=\frac{\sigma -\sigma_{P}}{\sigma_{s}-\sigma_{P}}$$

where *X_DRX_* is the dynamic recrystallized volume fraction; *σ_p_* and *σ_s_* are the peak stress and steady-state stress in stress–strain curves, respectively. The critical condition of dynamic recrystallization can be described as [16]:

(2) $$\theta ={\left(\frac{d\sigma }{d\varepsilon }\right)}_{\varepsilon ,T},\;\frac{\partial }{\partial \sigma }\left(-\frac{\partial \theta }{\partial \sigma }\right)=0$$

**Figure 5:**
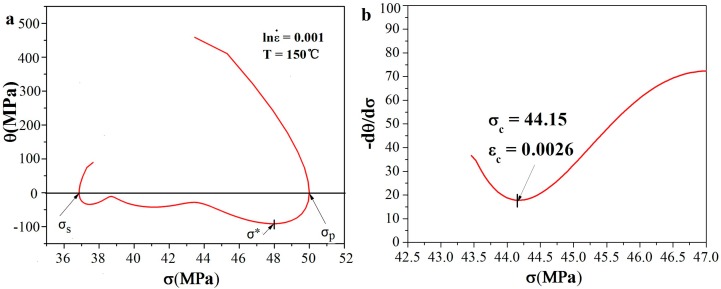
Relationship between *θ* and σ: (**a**) *θ* – *σ*; (**b**) −(*∂θ*/*∂σ*) − *σ*.

According to Figure 4 and Equation (2), the relationship between *θ* (*θ* = *dσ*/*dε* is strain hardening rate) and *σ* (flow stress) at the deformation temperature of 150 °C and strain rate of 0.001 s^−1^ can be calculated and shown in Figure 5a. While *σ_p_* (peak stress) is the first stress value when *θ* = 0, *σ_s_* is the second value. Thus, the *X_DRX_* can be calculated by Equation (1). 

For researching the relationship between *X_DRX_* and strain, the Avrami type equation has been demonstrated to describe the kinetic model of DRX [17,18]:

(3) $$X_{DRX}=1-\exp\left\{-k{\left[\frac{\varepsilon -\varepsilon_{c}}{\varepsilon_{p}}\right]}^{n}\right\}$$

*ε_c_* is the critical strain, *X_DRX_* is the volume fraction of dynamic recrystallization grain, and the minimum value in the −*dθ*/*dσ* versus *σ* curve in Figure 5b is the inflection point in the *θ* − *σ* curve in Figure 5a, which is the onset of DRX. According to Equations (1) and (3), *k* and *n* under different conditions can be calculated by regression analysis. The average values of k and n are determined to be −0.0686 and 1.4613. Thus, the kinetic model of DRX may be calculated using the following equation:

(4) $$X_{DRX}=1-\exp\left\{-0.0686{\left[\frac{\varepsilon }{\varepsilon_{p}}-0.519\right]}^{1.4613}\right\}$$

**Figure 6:**
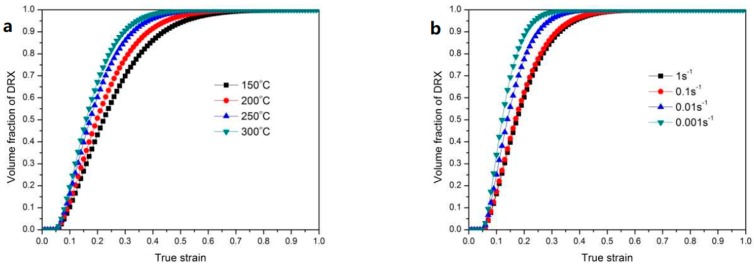
Predicted results of the relation between recrystallized volume fraction and true strain: (**a**) Strain rate = 1 s^−1^ with various temperatures; (**b**) T = 250 °C with different strain rates.

As shown in Figure 6, the kinetics behavior of dynamic recrystallization of this alloy can be described as S-curves of the recrystallized volume fraction expressed as a function of true strain calculated by Equation (4). It can be seen that the amount of DRX volume fraction increases with the increasing deformation strain and temperature or decreasing deformation strain rate. At the early part of the deformation stage, the DRX does not occur, the fraction of DRX increased sharply with the increasing deformation, and reached the completion of the DRX process.

**Figure 7:**
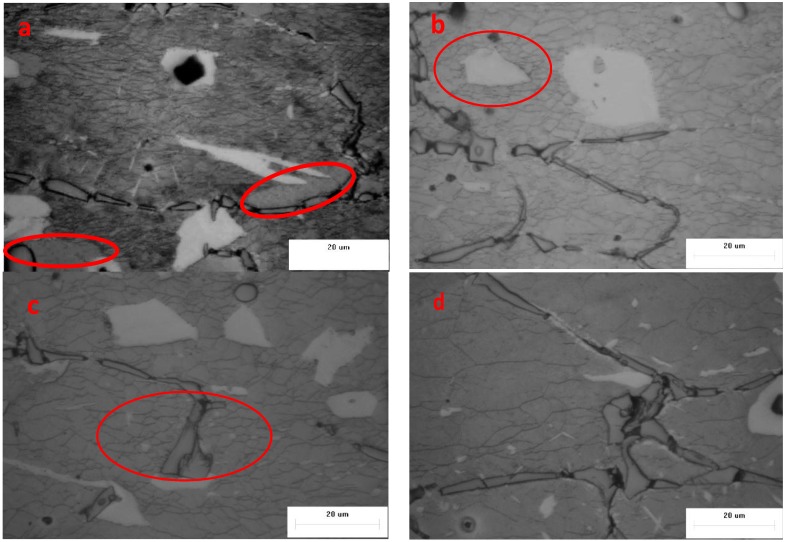
Optical microstructure of the material deformed at strain of 0.4 and (**a**) 150 °C, 1 s^−1^; (**b**) 200 °C, 1 s^−1^; (**c**) 250 °C, 1 s^−1^; (**d**) 300 °C, 1 s^−1^.

**Figure 8:**
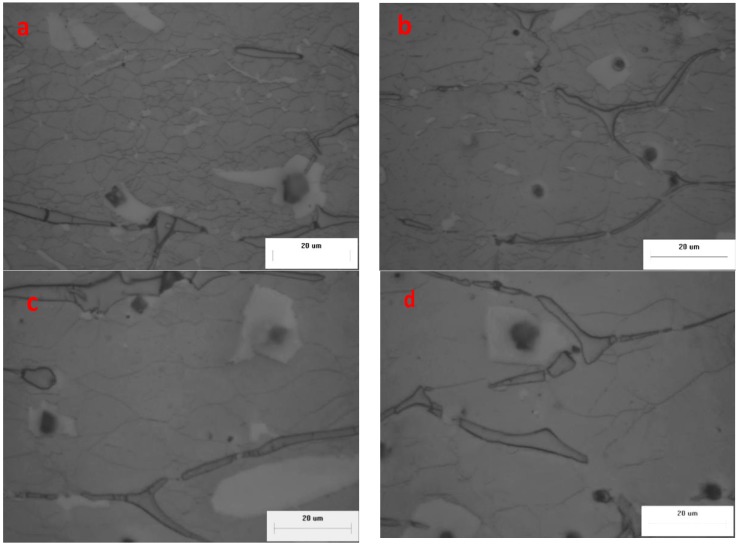
Optical microstructure of the material deformed at a strain of 0.4 and (**a**) 250 °C, 1 s^−1^; (**b**) 250 °C, 0.1 s^−1^; (**c**) 250 °C, 0.01 s^−1^; (**d**) 250 °C, 0.001 s^−1^.

As shown in Figure 7 and Figure 8, the effects of different deformation conditions on the DRX grain sizes at the strain of 0.4 were examined. As shown in Figure 7, the influence of different temperatures (150 °C, 200 °C, 250 °C, 300 °C) on the variation of recrystallized grain size at a steady state condition (strain rate = 1 s^−1^) was investigated under the optical microscope. The grains deformed along the compression direction and transformed to approximately fine equiaxed grains completely or partially. Compared with microstructure of the as-homogenized alloy, the recrystallized grains were obviously refined at the deformation conditions and the necklace or corrugated grain boundaries can be easily observed. It can be found that the grain size of specimens gradually becomes larger at higher temperature because of higher boundary mobility, which leads to dislocation generation and annihilation, further enhancing the nucleation and growth of DRX grains [19,20]. The dynamic recrystallization and dynamic recovery occur at high temperature deformation, which lead to flow stress softening. With the temperature increasing, higher velocity of grain boundary migration can be achieved, the grain size becomes larger and the stress softening is more obvious, which is conducive to reducing the work hardening caused by plastic deformation. In Figure 7a, there is no dynamic recrystallization in some places (the red circle) because the low temperature will retard the driving force of DRX. The fraction of DRX microstructure increases with increasing deformation temperature and the microstructure becomes more uniform due to coordination among grains during grain boundary migration. In Figure 7b, the DRXed grains of the matrix in the vicinity of the α-Mg phases are more refined than other ones. In Figure 7c, the same phenomenon can be observed in the red circle, the grains are refined around Al_4_Sr phases and a mixed distribution of particle sizes is presented. The local grain refinement around the secondary phases or particles was induced by local recrystallization [21]. In Figure 7d, the grain size of β-Li matrix is much bigger due to higher mobility of grain boundaries at 300 °C. Generally, owing to the physical properties differences of the β-Li matrix, α-Mg phases and the secondary particles, the dislocation piled up in the vicinity of hard phases and secondary particles, the complex dislocation arranging and storing energy increased in the local area, and the driving force for recrystallization increased, which can accelerate the recrystallization and increase the fraction of DRX via particle stimulated nucleation [22,23]. Thus, the fraction of DRX of this alloy increased sharply and reached the complete DRX during a short strain in crescent due to a mounting of α-Mg, Al_4_Sr and Al_2_Y phases. In contrast, for different strain rates of 0.001 s^−1^, 0.01 s^−1^, 0.1s^−1^ and 1 s^−1^, the microstructures of the specimens deformed to a strain of 0.4 at a fixed temperature of 250 °C are shown as Figure 8a–d, respectively. In addition, with the increasing of deformation strain rate, the Zener–Hollomon parameter (Z=εexp(Q/RT)) increased, the average grain size of specimens decreased due to increasing stored energy and decreasing grain boundary migration time [19]. Similarly, the lowest deformation temperature gives a higher value of Z, which gives refined and partially recrystallized microstructure with an average grain size of 2–3 µm (Figure 7a).

### 3.3. Corrosion Behavior

The dual-phase Mg-Li alloys have low density and better formality, but inferior corrosion resistance limits its rapid development and application. Generally, there are several influence factors that affect the corrosion resistance of Mg-Li alloys, the chemical composition, microstructure, surface treatment and application environment [24,25,26]. As a highly reactive element, Lithium was added to Mg alloys, which can further weaken the corrosion resistance of the Mg-Li matrix. Moreover, the duplex microstructures lead to local corrosion, which decrease the corrosion resistance of duplex phase Mg-Li alloy relative to single phase Mg-Li alloys [27]. The research of Song [4] indicated that phase boundaries are weak parts due to the inhomogeneous distribution of Lithium near the phase interface. It is well known that the β-Li phases with higher lithium content are more chemically and electrochemically active than α-Mg phases, and the corrosion micro-galvanic couples are formed on the surface of Mg-Li alloys, which accelerate the degradation of duplex phase Mg-Li alloys. Moreover, Song [10] reported that Lithium can affect the structures and compositions of the surface oxide film that are bound up with the corrosion resistance of the substrate. Thus, the corrosion protection of duplex phase Mg-Li alloys is more complex than the single phase ones.

It is reported that alloying, heat treatment and plastic deformation are simple ways to enhance the corrosion resistance of Mg-Li alloys. Due to the formation of oxide surface film with passivation properties, the addition of Al into Mg-Li alloys can improve the corrosion resistance of Mg-Li alloys. However, more researchers reported that the rare-earth (Ce and Y) can play a important role in enhancing the corrosion resistance [28,29]. Xu [29] reported that the continuous distributed I-phases are an effective way to obtain better corrosion resistance in Mg-Li alloys. Here, the Al_4_Sr phases have the same continuous network structure as I-phases in as-cast Mg-Li alloys, so it is necessary to ascertain the effect of Al_4_Sr phases on corrosion resistance of Mg-Li alloys.

First, the corrosion behavior of Mg-Li alloys needs to be figured out. The corrosion of duplex phase Mg-Li-Al alloy is a dissolution process, which contains a cathodic reaction and anodic reaction. The reaction equations are listed as follows [11,24]:

Mg → Mg^2+^ + 2e^−^, (5)

Al → Al^3+^ + 3e^−^, (6)

Li → Li^+^ + e^−^, (7)

2H_2_O + 2e^−^ → H_2_↑ + 2OH^−^. (8)

The equations of corrosion product formations are shown as follows:

Mg^2+^ + 2OH^−^ → Mg(OH)_2_↓, (9)

Al^3+^ + 3OH^−^ → Al(OH)_3_↓, (10)

Li^+^ + OH^−^ = LiOH↓. (11)

Moreover, the carbonates such as Li_2_CO_3_ and MgCO_3_ will form on the surface of Mg-Li alloy matrix.

**Figure 9:**
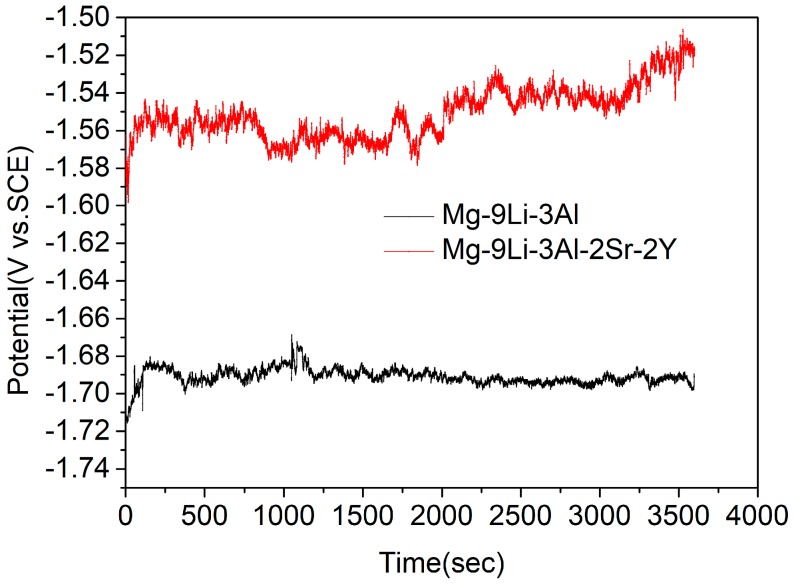
The open circuit potential (OCP)—Time curves of Mg-Li alloys in 3.5% NaCl solution.

As shown in Figure 9, the open circuit potential (OCP) of Mg-9Li-3Al and Mg-9Li-3Al-2Sr-2Y alloys are investigated. In the initial stage, the potentials are increasing rapidly. Then, the curves gradually become stable. The OCP of Mg-9Li-3Al-2Sr-2Y alloy is higher than Mg-9Li-3Al alloy obviously.

**Figure 10:**
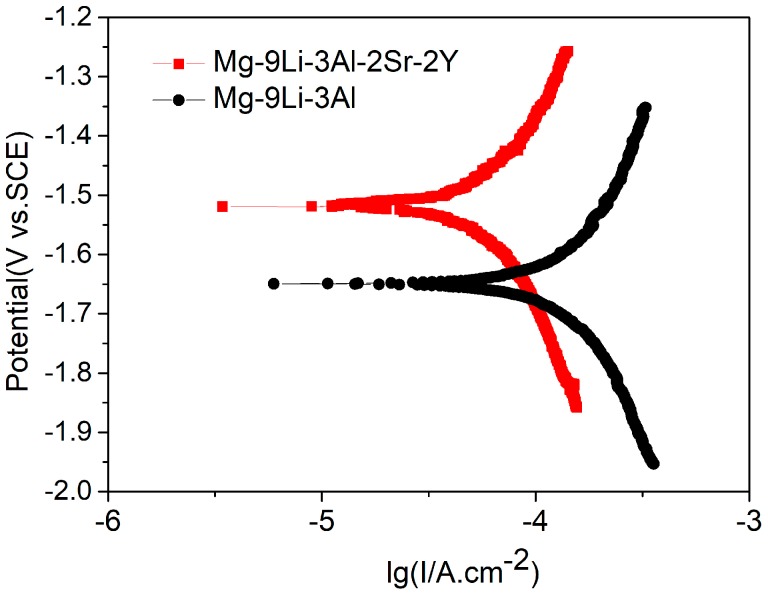
The polarization curves of Mg-Li alloys in 3.5% NaCl solution.

**Figure 11:**
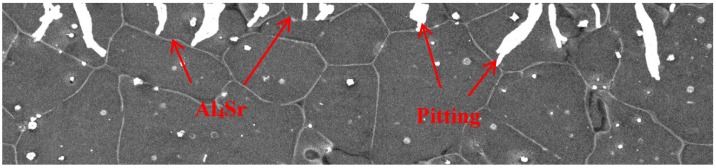
The schematic illustration of corrosion attack processes in Mg-9Li-3Al-2Sr-2Y alloy.

Through researching the corrosion behavior of Mg−8Li alloy in NaCl solution, Song [10] considered the corrosion behavior of Mg-Li alloys as filiform corrosion step by step. While the front of filament tip provides H+ and the hydrogen evolution reaction, the back end of the filament tip generates OH− and chemical precipitation, such as Mg(OH)_2_, Al(OH)_3_ and LiOH. Moreover, the addition of Li also aggravates the hydrogen evolution reaction. The matrices of Mg-Li alloys are eroded stage by stage. As the protective film preventing the matrix from corrosion, the carbonates and hydroxides’ corrosion products possess loose and porous structure, so the solution will corrode the matrix of Mg-Li alloy from the filiform gap [11,24]. However, after the addition of Sr, the continuous Al_4_Sr phases that were formed in the matrix acted as an effective barrier for protecting the Mg-Li matrix from pitting corrosion and filiform corrosion. Thus, the addition of Sr substantially shifts the polarization curve to more positive potentials compared to that of the Mg-9Li-3Al alloy (Figure 10), and it is confirmed that the Al_4_Sr phases with continuous network structure can prevent the Mg-Li alloys from further corrosion of infiltration solution (Figure 11).

## 4. Conclusions

In this work, the hot deformation behavior, dynamic recrystallization behavior and corrosion resistance of Mg-9Li-3Al-2Sr-2Y alloy were investigated, and the conclusions are as follows:

(1) The Mg-9Li-3Al-2Sr-2Y alloy presents obvious dual-phase structure, which is comprised of β-Li matrix, α-Mg phases with petal-like shape, continuous Al_4_Sr phases and globular Al_2_Y phases.
(2) The strain–stress curves are affected considerably by deformation temperatures and deformation strain rates. With the increasing of stress rate or decreasing of deformation temperature, the flow stress increased.
(3) The onset of DRX occurred before the peak stress, and the volume fraction of DRX grains under different deformation conditions were calculated by an Avrami type equation. With the strain increases, the DRX volume fraction increases and reaches completion of the DRX process.
(4) The Mg-9Li-3Al-2Sr-2Y alloy has better corrosion resistance than the Mg-9Li-3Al alloy. This phenomenon is attributed to the massive and continuous Al_4_Sr phase, which acts as a continuous barrier to protect the Mg-Li matrix.
